# The *Nix* locus on the male-specific homologue of chromosome 1 in *Aedes albopictus* is a strong candidate for a male-determining factor

**DOI:** 10.1186/s13071-018-3215-8

**Published:** 2018-12-24

**Authors:** Ludvik M. Gomulski, Marina Mariconti, Alessandro Di Cosimo, Francesca Scolari, Mosè Manni, Grazia Savini, Anna R. Malacrida, Giuliano Gasperi

**Affiliations:** 1 Department of Biology and Biotechnology “L. Spallanzani”, University of Pavia, Pavia, Italy; 2 Department of Genetic Medicine and Development, University of Geneva Medical School, and Swiss Institute of Bioinformatics, Geneva, Switzerland

**Keywords:** *Nix*, sex determination, sex chromosome, intron retention, virus vector, control strategies

## Abstract

**Background:**

Global concern over the rapid expansion of the Asian tiger mosquito, *Aedes albopictus*, and its vector competence has highlighted an urgent need to improve currently available population control methods, like the Sterile Insect Technique. Knowledge of the sex determination cascade is a prerequisite for the development of early-stage sexing systems. To this end, we have characterised the putative sex determination gene, *Nix*, in this species. In *Aedes* species the chromosome complement consists of three pairs of chromosomes. The sex determination alleles are linked to the smallest homomorphic chromosome.

**Results:**

We identified the male-specific chromosome 1 of *Ae. albopictus* that carries the putative male-determining gene *Nix*. We have also characterised the complete genomic sequence of the *Nix* gene which is composed of two exons and a short intron. The gene displays different levels of intron retention during development. Comparison of DNA sequences covering most of the *Nix* gene from individuals across the species range revealed no polymorphism.

**Conclusions:**

Our characterisation of the *Nix* gene in *Ae. albopictus* represents an initial step in the analysis of the sex determination cascade in this species. We found evidence of intron retention (IR) in *Nix*. IR might play a role in regulating the expression of *Nix* during development. Our results provide the basis for the development of new genetic control strategies.

**Electronic supplementary material:**

The online version of this article (10.1186/s13071-018-3215-8) contains supplementary material, which is available to authorized users.

## Background

The Asian tiger mosquito, *Aedes albopictus*, is among the most aggressive and invasive mosquitoes in the world. It is a vector of numerous pathogens including the world’s most prevalent arboviruses, dengue, chikungunya and Zika [1, 2]. *Aedes albopictus* has rapidly expanded from Southeast Asia to colonize both tropical and temperate countries and is now present in all continents [3–5] and has caused major chikungunya outbreaks in parts of Europe including Italy [6, 7] and France [8].

The global concern over the rapid expansion and the ability of *Ae. albopictus* to transmit infectious diseases has highlighted an urgent need to improve currently available population control methods [9]. These include the Sterile Insect Technique, which requires highly efficient early-stage sexing methods [10]. A better understanding of the sex determination mechanisms in *Ae. albopictus* would be thus of practical importance and would also benefit evolutionary studies of sex-specific sequences.

Unlike anophelines, the sex chromosomes are homomorphic in all culicine mosquitoes including *Aedes* species. In these species the chromosome complement consists of three pairs of chromosomes designated 1, 2 and 3 [11]. The sex determination alleles are linked to the smallest homomorphic chromosome 1 and are described as Mm in males and mm in females [12–14]. The homomorphic sex chromosome arrangement is considered the ancestral state of mosquito sex chromosomes [15]. Differences between the male and female sex chromosomes in the mitotic complements of nine *Aedes* species have been studied using Giemsa C-banding [16]. From the C-banding patterns, it was suggested that two separate lines of evolution for the chromosome 1 homologues have taken place, one leading to *Ae. aegypti* type and the other to the *Ae. albopictus* type [16]. However, as the C-banding pattern has been found to be polymorphic in different *Ae. aegypti* strains, clear sex chromosome differences were not well defined. In *Ae. aegypti* the development of integrated linkage, chromosome and genomic maps [17] were the premise for the identification of the homomorphic chromosome 1 M homologue and the location of a dominant male determining factor *Nix* in the M locus [18].

In *Ae. aegypti* the sex-determination locus resides in band 1q21 on the q-arm of chromosome 1, a region that is not subject to recombination [19]. Beyond this region recombination is thought to occur in an autosome-like fashion, maintaining the overall homomorphic structure of this chromosome [19]. Further genetic evidence has recently shown that chromosome 1 in *Ae. aegypti* is differentiated over a region much larger than the M-locus and shows features of an XY chromosomal system despite the apparent homomorphy [20].

A number of male-specific genomic sequences were identified in *Ae. aegypti* using the chromosome quotient method [18]. Among these sequences was a novel gene, *Nix*, that shared moderate identity with *transformer-2*, one of the key sex-determination genes in *Drosophila melanogaster* [21]. The *Ae. aegypti Nix* gene is 985 bp in length and encodes a 288 amino acid polypeptide with two RNA recognition motifs. *Nix* exhibits persistent male linkage and is expressed early in embryonic development [18]. These are essential characteristics of an M factor. Furthermore, somatic knockout of *Nix* in *Ae. aegypti* male embryos results in feminization, and ectopic expression of *Nix* in female embryos results in masculinization, clearly demonstrating that *Nix* is sufficient for the initiation of male development [18]. *Nix* is hypothesised to be a splicing factor that acts directly or indirectly on the *doublesex* (*dsx*) and *fruitless* (*fru*) genes involved in sex-determination [22].

In *Ae. albopictus* the M and m homomorphic homologues of chromosome 1 have not been identified. Hall et al [18] identified an incomplete *Nix* homologue in *Ae. albopictus* (GenBank accession number KP765684). Recently, the complete *Ae. albopictus Nix* sequence was identified from a C6/36 cell line genome [23]. This complete sequence is almost identical to the *Nix* sequence that we independently identified in the *Ae. albopictus* Rimini strain and that we describe both cytologically and functionally in this paper.

Using morphological, cytogenetics and molecular approaches, we have identified the male-specific chromosome 1, that carries the putative male-determining gene, *Nix*. This gene has all the characteristics of a dominant, male-determining factor. Characterisation of *Nix* in *Ae. albopictus* revealed a possible mechanism (intron retention, IR) that regulates its expression during embryogenesis and development. Our findings may be applied to further understanding of the sex-determination cascade and the development of novel genetic control strategies that can help combat this important disease vector by converting females into harmless males. Furthermore, we have developed markers useful for sexing pre-imaginal *Ae. albopictus* individuals from the embryo to the pupal stage.

## Methods

### Mosquitoes

The Rimini laboratory strain was used. This strain was established in 2004 from eggs collected in ovitraps from Rimini, Emilia Romagna, Italy (44°03'24"N, 12°33'52"E) [24]. The strain has been maintained in Pavia since its arrival in December 2013 at generation 53 from Romeo Bellini of the Centro Agricoltura Ambiente “G. Nicoli” in Crevalcore (Italy). The strain is maintained at 26 °C with 70 % relative humidity and a 12:12 h (light:dark) photoperiod. Larvae are reared on fish food pellets (Tetra Goldfish Granules), whereas adults are maintained on 20 % sugar solution and commercially available pig or cow blood using a membrane feeding apparatus.

Samples collected from wild populations were also used. These included individuals from northern Italy (Arco in Trento, collected in August 2012), Croatia (Velika Gorica, collected in August 2017), Réunion (St Denis, collected in March 2010), China (Guangzhou, collected in November 2017) and two from Thailand (Ban Rai and Chiang Mai, both collected in November 2010). The Thai Chiang Mai sample consisted of DNA extracted from a pool of individuals.

Pupae were sexed using the sexual dimorphism of the tenth abdominal segment or genital pouch [25].

### Isolation of developing gonads in *Ae. albopictus* larvae

Fourth-instar larvae were dissected to isolate the developing gonads following the protocol described for *Ae. aegypti* with modifications [26]. Briefly, individual fourth-instar larvae were transferred to a drop of phosphate-buffered saline (PBS) on a glass slide and the head removed. Using fine-tipped forceps, the abdomen was laterally incised at the level of the sixth segment and the cuticle removed to expose the gonads. The gonads were then transferred in a drop of PBS on clean glass slide, covered with a coverslip, and observed under a Zeiss Axioplan microscope at 200× and 400× magnification. Images were captured using an Olympus DP70 digital camera.

### Nucleic acid preparation and sequence analyses

For large individuals, such as larvae, pupae and adults, the specimen was divided in two parts transversally and the abdomen used for RNA extraction using TRIzol Reagent (Invitrogen) following the manufacturer’s protocol. The head and thorax were used for DNA extraction [27]. Likewise, for large egg collections, the sample was divided into two parts for DNA and RNA extraction. RNA was also extracted from different body compartments of 2-4 day-old males using TRIzol using the following pools: antennae (~100 pairs), palps (~100 pairs), proboscises (25), heads without antennae, palps and proboscises (5), legs (~10 sets), thoraces without wings and legs (5), abdomens (5) and wings (50 pairs). RNAs were treated with DNAse I (DNAfree, Ambion), whereas DNAs were treated with RNase A (Invitrogen). The nucleic acids were resuspended in DEPC-treated TE buffer (10 mM Tris-HCl, pH 8, 1 mM EDTA) and concentrations were determined using a Nanodrop ND-1000 spectrophotometer (Nano-drop Technologies Inc, Wilmington, DE, USA).

PCR amplifications were performed using *Taq* DNA polymerase or AccuPrime *Taq* DNA Polymerase High Fidelity Kit (Invitrogen) using the following cycle conditions: 95 °C for 2 min, 35 cycles at 95 °C for 20 s, 58 °C for 20 s, 72 °C for 1 min, and a final extension at 72 °C for 5 min for the Nix-309, Nix-833 and 18S rDNA primers (Additional file 1: Table S1). The extension time was increased to 1 min 30 s for the Nix-1121 primer pair. The 18S rDNA fragment was amplified as a control for DNA integrity. Amplification products were electrophoresed on 1.5 % agarose gels and sequenced directly or cloned using the TOPO TA cloning kit (Invitrogen) before sequencing both strands (Macrogen Europe).

Synthesis of cDNA was performed using 200 ng RNA (with the exception of single embryos where the complete RNA sample was used) in 20 μl reaction volumes using the iScript^TM^ cDNA Synthesis Kit (Bio-Rad). RT-PCRs with gene-specific primers, designed using Primer3Plus [28] (Additional file 1 : Table S1), were performed using 5 % of the synthesized cDNA and the cycle conditions were those used for DNA amplifications. An 18S rDNA fragment was amplified as a control for cDNA integrity. To check for genomic DNA contamination, RT-PCR was also performed on controls, i.e. RNA samples in which cDNA synthesis had been performed in the absence of reverse transcriptase. Amplification products were electrophoresed on 1.5 % agarose gels. The amplification products were cloned and sequenced as above.

Sequence analyses were performed using the BLAST family of programs [29, 30] and Conserved Domain search [31] from the National Centre for Biotechnology Information. Sequences were aligned manually or using CLC Main Workbench 6.9.1 (CLC bio).

### Metaphase chromosome preparation and *in situ* hybridization

Mitotic chromosome spreads were obtained from the imaginal discs of sexed fourth-instar larvae (Rimini strain) following the protocol of Sharakhova et al. [32]. Briefly, for each larva, the thorax, previously isolated in PBS, was transferred to cold hypotonic solution (1 % sodium citrate) and dissected to extract the imaginal discs. After incubation in 1 % sodium citrate for 10 min at room temperature the imaginal discs were transferred to methanol-acetic acid 3:1 solution for 4 min. Subsequently, 100 μl 60 % acetic acid was added to the material for chromosome fixation and the disrupted imaginal discs were dropped onto clean slides and dried. The slides were stained with DAPI (4′,6-Diamidine-2′-phenylindole dihydrochloride; 10 ng/ml in 4xSSC) which produces a pattern similar to Hoechst 33258, which in *Drosophila* stains heterochromatic regions [33].

Fluorescence *in situ* hybridizations of *Nix* and 18S rDNA probes were performed on mitotic chromosome preparations obtained from sexed larvae. An 833 bp *Nix* sequence cloned in pCR^TM^ 2.1-Topo vector, and a 757 bp 18S rDNA PCR fragment were used as probes. The probes were labelled using the Biotin High Prime kit (Roche) and detection of hybridization signals was performed using the Alexa Fluor 594 Tyramide Signal Amplification Kit (Invitrogen). Chromosomes were counterstained and mounted using the VECTASHIELD mounting medium (Vector Laboratories, Burlingame, CA, USA). Hybridization and DAPI fluorescence signals were visualized through appropriate filters using a Zeiss Axioplan microscope. Images were captured using an Olympus DP70 digital camera with exposure times of 0.5 and 0.2 s for rhodamine and DAPI, respectively.

The karyotype description [34] was adopted for the chromosomal localization of *Nix* and 18S rDNA on mitotic chromosomes.

## Results

### Sexing of fourth-instar larvae through dissection of developing gonads

**Fig. 1:**
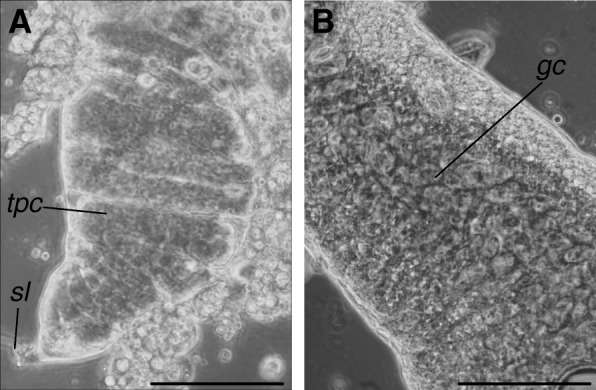
Gonads of fourth-instar larvae. **a** Developing testes showing transverse pseudochambers (*tpc*), which represent the spermatic tubes, and the suspensorial ligament (*sl*). **b** Developing ovary showing the germ cells (*gc*). *Scale-bars*:100 μm

Larvae were sexed in order to obtain sex-specific mitotic chromosome spreads from the imaginal discs. The developing gonads in *Ae. albopictus* fourth-instar larvae displayed a clear-cut sexual dimorphism that allowed us to sort male and female larval stages. Specifically, the developing testes, located ventrally in the sixth larval abdominal segment, display a pyriform shape and appear to be divided into transverse pseudochambers, which represent the spermatic tubes with the germ cells irregularly arranged (Fig. 1a). A short filament, i.e. the suspensorial ligament, is visible at the anterior end. The ovaries are fusiform and are located lateroventrally to the dorsal tracheal trunks in the sixth abdominal segment. They contain irregularly distributed round germ cells (Fig. 1b).

### Chromosome 1 homologues display sex-related features

Metaphase chromosome preparations from male and female larvae of the Rimini strain portray a clear and reproducible karyotype with three pairs of chromosomes which, according to the nomenclature of McDonald & Rai [34], and by analogy to the *Ae. aegypti* karyotype [32], were designated as chromosomes 1, 2 and 3. Apart from the metacentric chromosome 3, chromosomes 1 and 2 fall in the sub-metacentric category [11].

**Fig. 2:**
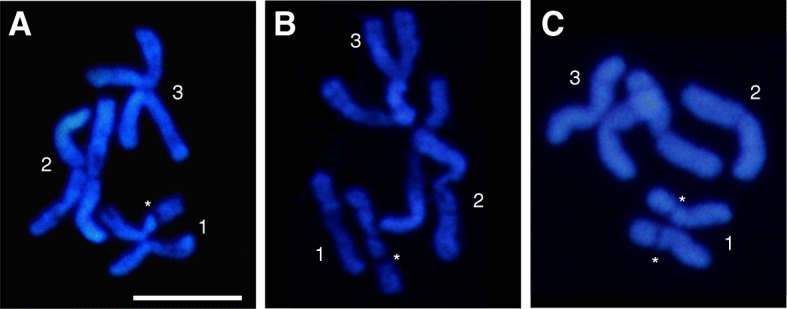
DAPI stained mitotic chromosomes. **a**, **b** Male karyotype showing a DAPI-negative band (asterisk) on only one homologue of chromosome 1. **c** Female karyotype displaying DAPI-negative bands (asterisks) on both homologues of chromosome 1. *Scale-bar*: 5 μm

DAPI staining of the mitotic chromosome complements from several sexed larvae shows consistent differences in the banding patterns between the chromosome 1 homologues in males and females (Fig. 2). In females, both the smaller arms (p-arm) of the chromosome 1 homologues display a DAPI-negative band which, in males, is present only on one of the homologues. The same sex-related banding pattern on chromosome 1 was obtained with Giemsa C-staining (data not shown). On this basis, we further investigated whether the male-specific chromosome 1 homologue lacking the DAPI-negative band might be related to maleness/male differentiation. Considering that a male determining gene, *Nix*, has been mapped on chromosome 1 in *Ae. aegypti* [18], we searched for the presence of a *Nix* orthologous sequence on this male chromosome in *Ae. albopictus*.

### A *Nix* orthologous sequence is present in the *Ae. albopictus* genome

We used a previously identified 701 bp partial *Ae. albopictus Nix* cds [18] (GenBank: KP765684.1) as a BLASTN query against the *Ae. albopictus* genome sequence from the Fellini aka Rimini strain [24]. This resulted in the identification of two contigs (C8454750: 379 bp and C5613352: 257 bp; Additional file 2) that partially overlap the query sequence with 100 % identity at both the 5' and 3' ends, extending the *Nix* sequence by 174 bp and 145 bp at the 5' and 3' ends, respectively. The resulting composite sequence of 1020 bp in length (Additional file 3: Figure S1) was then used as a BLASTN query against a transcriptome assembled with Trinity [35] derived from Rimini strain male and female antennae at different physiological states (Gomulski et al., unpublished data). Four transcripts sharing 100 % identity with the *Nix* sequence (comp292865_c0_seq1: 583bp; comp292865_c0_seq3: 214 bp; comp770372_c0_seq1, 257 bp and comp618873_c0_seq1: 603 bp; Additional file 2) were identified which extended the 3' end of the *Nix* sequence by 560 bp, resulting in a composite sequence of 1580 bp in length (Additional file 3: Figure S1). Finally, this composite sequence was used as a BLASTN query against the Fellini genome, which identified an additional contig (C7387974: 315 bp; Additional file 2) with 100 % identity. As the 1580 bp *Nix* composite sequence completely encompasses the Fellini genomic contig C7387974 sequence the *Nix* locus was not extended (Additional file 3: Figure S1). BLASTX analysis of the obtained 1580 *Nix* sequence against the non-redundant database gave, as expected, a perfect hit to the previously identified partial *Ae. albopictus* AKI28880.1 NIX protein [18]. In addition, a 171 bp region downstream of that hit also gave 57/72 % identity/similarity to the C-terminal part of the *Ae. aegypti* NIX protein (AHW46195.1), suggesting that the 1580 bp sequence contains the complete coding sequence. This 1580 bp *Nix* composite sequence shares 99.9 % identity with a region on a 971 Kb contig (MNAF02001502.1) present in the *Ae. albopictus* C6/36 cell line genome [23] (GCA_001876365.2 assembly) and predicted to encode a 282 aa NIX protein. The only difference between the sequences was an 8 bp deletion in the MNAF02001502.1 sequence 56-64 upstream of the *Nix* start codon.

**Fig. 3:**
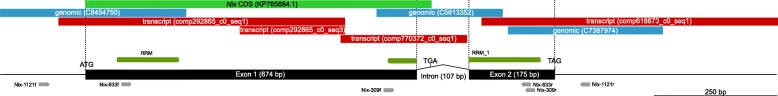
Scheme of the characterization of the *Nix* locus *in silico* combining genomic and transcriptome sequences to derive a composite sequence. The stop codons in the intron-retaining transcript and spliced transcript are indicated. The positions of the three pairs of primers and the two RNA recognition motifs (RRM) are shown

Examination of our composite sequence indicated that the *Ae. albopictus Nix* gene consists of two exons of 674 bp and 175 bp separated by a 107 bp intron flanked by donor/acceptor sites. The 5' donor splice site is AG^GUUUGU rather than the canonical AG^GUAAGU sequence for *Ae. aegypti* [36]. The position of the intron is conserved with respect to *Ae. aegypti Nix* (NCBI gene report *Nix*/LOC110678376). The *Ae. albopictus Nix* gene is predicted to encode a protein of 282 aa with two RNA recognition motifs (RRM smart00360 from 21-63aa and RRM_1 pfam00076 from 205-273aa) (Fig. 3).

### *Nix* is male-linked and is located on the male chromosome 1 homologue

**Fig. 4:**
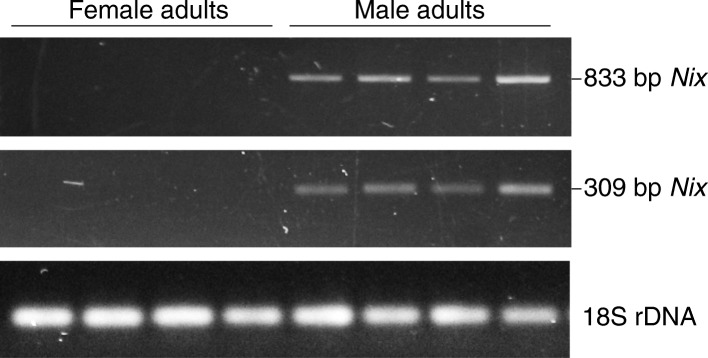
Amplification of 833 bp and 309 bp *Nix* fragments from genomic DNA. Amplification of a 18S rDNA fragment in both sexes as a control for DNA integrity

The use of two primer sets spanning the *Nix* intron (Nix-309f/r and Nix-833f/r; Fig. 3; Additional file 1: Table S1; Additional file 3: Figure S1) on genomic DNA, derived from male and female adults from different generations of the Rimini strain, produced amplification products only in males (Fig. 4). The sequences of these amplicons were identical to the *in-silico* sequence with the expected sizes of 309 and 833 bp, respectively. The 833 bp sequence includes 602 bp of exon 1, the 107 bp intron, and 124 bp of exon 2, while the 309 bp sequence includes 69 bp of exon 1, the 107 bp intron, and 133 bp of exon 2. Moreover, a 1121 bp sequence of a cloned fragment resulting from amplification of male genomic DNA with a third primer pair spanning the entire coding sequence (Nix-1121f/r; Fig. 3; Additional file 1: Table S1; Additional file 3: Figure S1) showed complete identity with the *in-silico* sequence and supported the predicted *Nix* gene structure.

**Fig. 5:**
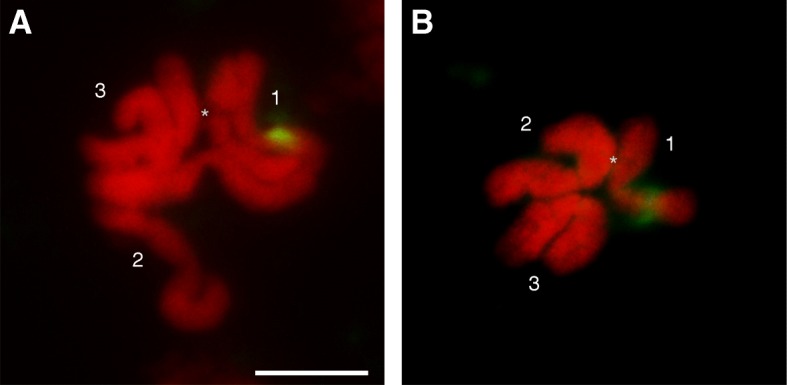
Fluorescence *in situ* hybridization (FISH) to mitotic chromosomes using a *Nix* fragment as probe (**a**) and a 18S rDNA fragment as probe (**b**). The three pairs of chromosomes are less evident in these images due to somatic pairing [63]. The asterisk indicates the position of the DAPI-negative band on one homologue of chromosome 1. *Scale-bar*: 5 μm

Fluorescence *in situ* hybridization (FISH) on mitotic chromosomes from male and female sexed larvae, using the 833 *Nix* fragment as a probe, allowed the localization of *Nix* to a single position on only one homologous copy of chromosome 1, the male chromosome, i.e. to the homologue that lacks the DAPI-negative band on the p-arm. Specifically, the *Nix* signal localizes on the q-arm of the male chromosome (Fig. 5a). On the same arm (q) of both homologues of the chromosome 1 pair is located the 18S ribosomal gene as demonstrated by FISH using a 757 bp 18S rDNA fragment probe (Fig. 5b).

### *Nix* displays very early male transcription during embryogenesis with intron retention

**Fig. 6:**
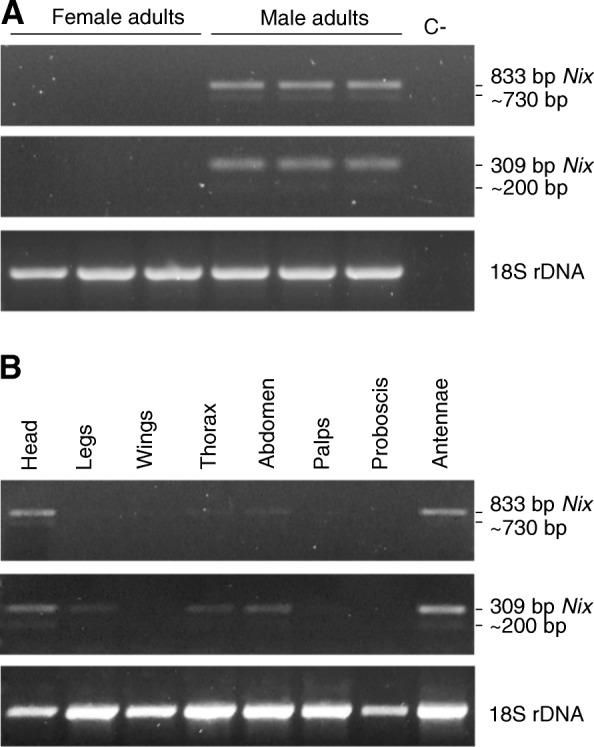
RT-PCR amplification of 833 bp and 309 bp *Nix* fragments from male and female cDNA derived from whole individuals (**a**), and different body compartments of male individuals (**b**). Amplification of a ribosomal 18S fragment was used as a control for cDNA integrity. The negative control (C-) without cDNA template is indicated

**Fig. 7:**
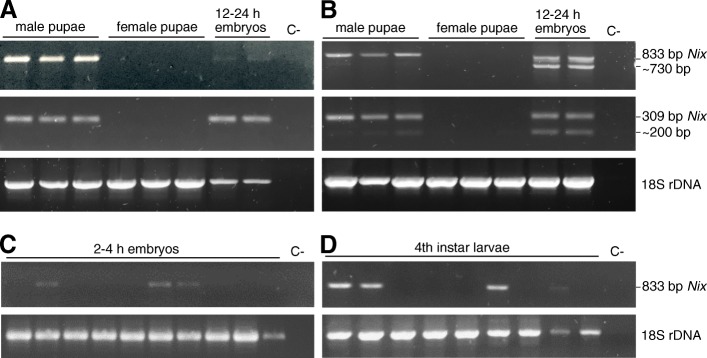
**a** DNA amplification of 833 bp and 309 bp *Nix* fragments from the head/thorax of male and female pupae and from 12-24 h embryo pools. **b** RT-PCR amplification of 833 bp and 309 bp *Nix* fragments from cDNA derived from the abdomens of the same male and female pupae in **a**, and cDNA from 12-24 h embryo pools. **c** RT-PCR amplification of 833 bp *Nix* fragments from cDNA derived from individual embryos processed 2-4 h after deposition. **d** RT-PCR amplification of 833 bp *Nix* fragments from cDNA derived from sexed individual fourth-instar larvae (lanes 1,2,6 and 8 are male larvae). The negative control (C-) without cDNA template is indicated. Amplification of a ribosomal 18S fragment was used as a control for DNA/cDNA integrity

The same set of primers previously used for *Nix* DNA amplification (Nix-833f/r and Nix-309f/r) were used for RT-PCR amplification of cDNA derived from single male and female adults. *Nix* amplicons were consistently obtained only in males (Fig. 6a) and appeared to have the same molecular weight as those obtained from genomic DNA. Cloning and sequencing of Nix-833f/r amplicons confirmed that the *Nix* transcript was 833 bp in length (like the DNA amplicon) and that it retained the intron sequence. These transcripts are predicted to encode a truncated protein of 233 aa due to the presence of an in frame stop codon within the retained intron sequence (Fig. 3). *Nix* transcripts were detected in different body compartments including the head, antennae, thorax, abdomen and legs, although the transcript appeared to be more abundant in the head and antennae (Fig. 6b), which is in agreement with Matthews et al. [37] who found highest *Nix* transcript abundances in male brain and antenna tissues of *Ae. aegypti*. Again, in these body compartments the size of the *Ae. albopictus Nix* transcript was that obtained from genomic DNA, although in the samples with higher transcript abundances, such as head and antennae, a very faint lower molecular weight band was discernible, corresponding to the putative spliced form. During the different developmental stages, starting from 2-4 h single embryos, to larvae and pupae (Fig. 7b, d, c, respectively), the *Nix* male-biased transcription is evident with the two different molecular weight transcripts displaying different relative abundances. This is very evident in 12-24 h embryos where the lower molecular weight fragment appears to be as abundant as the higher molecular weight fragment (Fig. 7a, b). The sequences of the cloned Nix-309f/r RT-PCR amplification products from these 12-24 h embryos confirmed that these bands represent the 309 bp unspliced intron-retaining *Nix* transcript fragment and the 202 bp spliced *Nix* transcript fragment (Additional file 4: Figure S2).

### *Nix* sequences do not display variability in natural populations of *Ae. albopictus*

A total of 11 direct Nix-833r/f PCR product sequences were obtained from male mosquitoes collected across the distribution range of *Ae. albopictus*. Six were from individuals from the home range area: Guangzhou, China, Ban Rai and Chiang Mai in Thailand, while five sequences were derived from two adventive Mediterranean populations, Arco in North Italy and Velika Gorica in Croazia. The partial sequences obtained from these individuals range from 720 bp (Guangzhou) to 767 (Arco) and include the intron sequence. The sequences aligned to the Rimini *Nix* reference sequence show no polymorphisms (Additional file 5: Figure S3).

## Discussion

The most important findings of the present study are (i) the identification in *Ae. albopictus* of the male-specific chromosome 1 homologue, which carries the putative male-determining gene, *Nix*; (ii) *Nix* displays precocious and male-specific transcription during development; (iii) *Nix* displays intron retention within the mature transcript; (iv) the *Nix* sequence does not appear to be polymorphic in populations across the species range, and (v) markers to sex *Ae. albopictus* from the single embryo to the pupa have been developed.

### The male-specific chromosome 1 homologue contains a potential male determining sequence, *Nix*

The protocol that we describe for sexing fourth-instar larvae facilitates the isolation and analysis of the male and female mitotic metaphase chromosome complements. As a result, the male-specific homomorphic chromosome 1 has been recognised through the absence of a DAPI-negative band located about halfway along its p-arm. This band represents a reproducible landmark for this chromosome and for the recognition of the p-arm. This cytological marking system is based on the karyotype present in the long-established reference laboratory strain, Rimini, derived from mosquitoes of an adventive Italian population. The Fellini genome sequence was derived from this strain [24]. Considering the great variation in terms of genome size and repetitive sequences detected in strains from different regions [38–42] we cannot exclude that naturally occurring variation in the distribution and amount of heterochromatin might affect the chromosomal banding pattern, and consequently the marking system of the male chromosome 1 in different strains/populations. Such assessment/evaluation is prevented by the lack of refined chromosome idiograms in this species. Nevertheless, the Giemsa C-banding technique applied to different strains from different origins showed the invariable presence on chromosome 1 of an intercalary band which, in males, is present only on one homologue [16, 43]. Also, in *Ae. aegypti*, Giemsa C-banding demonstrated differences between the male and female chromosome 1 homologues. *Aedes aegypti* females have a pericentromeric and an intercalary band, both of which may be absent on one of the chromosome 1 homologues in males. However, in this species the banding pattern has been found to be variable in different strains, both for the presence/absence and sizes of the intercalary bands in both males and females [16, 44, 45]. Indeed, physical chromosome-based map analyses demonstrated that the homomorphic chromosome 1 of *Ae. aegypti* had an evolutionary higher rate of genomic rearrangements than the autosomes, with minisatellites playing the major role in the rapid evolution [17].

**Fig. 8:**
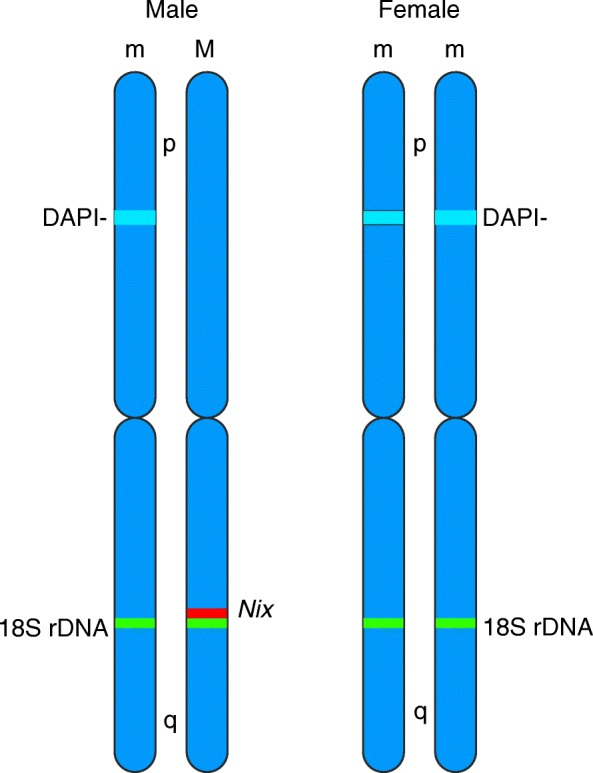
Schematic representation of the positions of *Nix*, 18S rDNA and of the sex-related DAPI-negative band (DAPI-) on chromosome 1 of male and female individuals. The relative positions of the *Nix* and 18S rDNA loci were determined visually from the corresponding individual *in situ* hybridisations

Using our cytological marking system as a physical anchor point, we located the male-specific *Nix* sequence almost halfway along the q-arm of the male-specific chromosome 1 homologue of the Rimini strain. *Aedes albopictus Nix*, like its *Ae. aegypti* orthologue, has the attributes to be considered as a male-determining factor [18]. It exhibits persistent male-specific linkage and expression and, more importantly, it displays very early embryonic expression. Indeed, *Nix* transcription is visible in 2-4 h single embryos. This is the stage that precedes blastoderm formation (at 5-6 h [46]), before sex is established. The functional homology between the *Ae. albopictus* and *Ae. aegypti Nix* sequences is further supported by their chromosomal locations; they are both located roughly on the same region of the q-arm of chromosome 1 in synteny with the 18S ribosomal genes. Synteny conservation and consequently homology between these two chromosomes has been identified using RFLP comparative mapping [47]. Given this background, we can consider the male chromosome 1 carrying *Nix* as M and the other copy without it as m, following the terminology of Motara and Rai [16] (Fig. 8). It remains to be assessed whether *Ae. albopictus Nix* resides in a non-recombining region (sex-determining region, SDR) like *Nix* in *Ae. aegypti* [18–20] and the extent of the SDR. This information will provide valuable data on the presence and extent of genetic differentiation between the two chromosome 1 homologues.

### Intron retention (IR) in *Nix* transcripts: a mechanism to modulate gene expression during development?

Another feature we found in *Nix* transcription, i.e. intron retention, may act as a mechanism to modulate its activity. In the different developmental stages, the *Nix* intron sequence is retained in a variable proportion of transcripts. Intron retention (IR), a type of alternative splicing, is the complete retention of an intron in a mature transcript. Transcripts containing retained introns can have different fates, they may undergo exosome-mediated degradation within the nucleus, or, if they arrive in the cytoplasm, they can give rise to truncated proteins or be degraded by nonsense-mediated decay [48]. IR is likely to be a cellular mechanism to modulate gene expression levels [49–51].

Retained introns tend to be short compared to other introns and have weak 5' and 3' splice sites [48]. The *Ae. albopictus Nix* intron is short at 107 bp and has a non-canonical 5' donor splice site. The *Nix* 5' donor splice site is AG^GUUUGU rather than the canonical AG^GUAAGU sequence for *Ae. aegypti*, whereas the *Nix* 3' acceptor splice site (UUUCAG^GU) corresponds to the canonical *Ae. aegypti* sequence, UUNCAG^GU [36]. Retention of the intron in *Ae. albopictus Nix* may thus be a result of inefficient recognition of the 5' splice site [48, 52]. That the efficiency of splicing of the intron appears to vary in different body compartments and in different developmental stages is interesting, as intron retention has been shown to be more common in transcripts that are less required for the physiology of a particular tissue [53]. Of all the developmental stages that we analysed, the most evident presence of the spliced *Nix* isoform was in the 12-24 h embryos, where the spliced and intron-retention forms appeared to be almost equally abundant (Fig. 7b). This developmental range, in *Ae. aegypti*, covers a very rapid embryonic segmentation kinetics which includes the onset of gastrulation (12 h), initiation of segmentation and ventral cord formation (15 h), labral lobe formation (18 h) and anterior and posterior midgut invagination formation (19 h) [46]. The fainter bands, corresponding to the spliced isoform, visible in male pupae (Fig. 7b), whole adult males (Fig. 6a) and some adult male body compartments especially the head and antennae (Fig. 6b), suggest that some splicing is occurring throughout the tissues or, at least, in a subset of cells.

The *Ae. albopictus* NIX protein has a moderate, partial identity (39 %) with *D. melanogaster* Transformer 2 protein (TRA2), one of the key genes in the sex-determination pathway. TRA2 inhibits the splicing of the M1 intron of its own *tra2* pre-mRNA. The resulting transcripts encode a non-functional protein that lacks one of the two regions rich in arginine and serine (RS domains) that facilitate protein-protein interactions critical in the regulation of alternative splicing. These M1 intron-retaining *tra2* RNAs are found almost exclusively in the male germline where they constitute 50 % of *tra2* mRNA. M1 retention has been shown to be a negative-feedback mechanism by which the functional TRA2 limits its own synthesis by promoting the accumulation of M1-containing transcripts. The absence of this negative-feedback mechanism in the *Drosophila* male germ line results in defective spermatogenesis [49, 54].

In *Ae. albopictus*, the retention of the intron in the mature *Nix* cDNA is predicted to result in a truncated protein of 233 aa due to the presence of a stop codon within the intron sequence. Just as the *Drosophila* truncated TRA2 proteins encoded by the IR transcripts lack an RS domain, the truncated *Ae. albopictus* IR NIX proteins lack one of the two RRMs. Like TRA2, NIX in *Ae. aegypti*, has been hypothesized to be a splicing factor that is responsible, directly or indirectly, for the male-specific splicing of *dsx* and *fru* [22]. This male-specific splicing of these two genes, which are important components of the sex-determination pathways of many insects, triggers cascades that give rise to the development of sexually dimorphic traits [55]. Indeed, silencing of *Nix* results in female splice variants of *dsx* and *fru* in male *Ae. aegypti* and morphological feminization, and ectopic expression of *Nix* in female embryos results in masculinization of the resulting adults [18]. The roles of the multiple *tra-2* homologues in *Aedes* mosquitoes are not clear. Knockdown of three of the four *tra-2* paralogues in *Ae. aegypti* did not alter *dsx* splicing or cause sex conversion of the resulting adults [55]. One of the *tra-2* paralogues has however been shown to be implicated in sex ratio distortion in *Ae. aegypti* apparently due to segregation distortion that affects gametic function, and female-specific zygotic lethality [56].

### The *Nix* sequence is invariable in populations

Despite the high degree of genetic variability present in native Asian *Ae. albopictus* populations and maintained in adventive populations [4, 57], the *Nix* sequence, covering most of the two exons and including the short intron (720-767 bp), shows no nucleotide variability in the populations analysed. *Nix* has a very short intron and encodes a small protein: this condition is expected considering its putative male-determining function and its very early zygotic expression [46]. In *Drosophila*, the majority of early zygotic expressed genes are intronless or have small introns and encode small proteins [58, 59]. *Nix* transcription in *Ae. albopictus* has been detected before the syncytial blastoderm stage, where several waves of rapid mitotic cell division occur and when the transcription of zygotic genes begins. During these rapid mitotic cycles the time required to transcribe genes with long primary transcripts is not trivial and poses constrains in terms of cost to their expression [58, 60]. Indeed, in *Drosophila* long zygotically expressed genes show delayed expression compared to shorter ones. This delay results from the inability to completely transcribe their long transcripts. It has been suggested that highly expressed early zygotic genes are subject to purifying selection to maintain short transcript length [61]. Selection may be acting in limiting the expansion of the *Ae. albopictus Nix* intron to preserve efficient transcription in the embryo. A similar lack of polymorphism was reported in the Y-linked gene *Dhc-Yh3* in lines of *D. melanogaster* and *D. simulans* [62]. Numerous factors can help interpret the lack of variation of these genes, such as the absence of recombination, codon bias and selective sweeps.

## Conclusions

Here we provide basic information essential to improve existing technologies, involving the rearing and release of large numbers of *Ae. albopictus* mosquitoes, to eliminate or modify its populations. The characterization of the male and female mitotic karyotypes and the identification of the male-specific chromosome 1 homologue that carries the putative male-determining gene, *Nix*, are essential prerequisites for the development of any genetic sexing system. They are a premise for the development of male and female physical maps and genome analyses in *Ae. albopictus*. This will facilitate the identification of genomic traits controlling sex determination/differentiation, reproduction, development, physiology and those related to susceptibility/refractoriness to different pathogens. The characterisation of the *Nix* gene in *Ae. albopictus* represents an initial step in the analysis of the sex determination cascade in this species: we have identified a possible mechanism that regulates its expression in embryogenesis and during development. This provides insights for developing new genetic strategies that convert female mosquitoes into harmless males. Finally, using morphological, cytogenetics and molecular approaches we have developed markers which are useful for sexing pre-imaginal *Ae. albopictus* individuals from the embryo to pupal stage.

## Additional files

Additional file 1: **Table S1.** Primer sequences. (DOCX 12 kb)

Additional file 2: **Text.** Genomic (Fellini aka Rimini) and transcriptome (Rimini) sequences used in the assembly of the *Ae. albopictus Nix* locus. (TXT 2 kb)

Additional file 3: **Figure S1.** (PDF) Sequence of the *Nix* locus showing the positions of the exons (green box), intron and predicted amino acid sequence. The positions of the primers are shown. (PDF 76 kb)

Additional file 4: **Figure S2.** (PDF) Alignment of the 12-24 h embryo-derived *Nix* transcript sequences showing a transcript with intron retention (unspliced) and a spliced transcript. The predicted translation products of the fragments are shown. (PDF 35 kb)

Additional file 5: **Figure S3.** (PDF) Alignment of the 833 bp *Nix* fragment from the Rimini reference sequence with the 10 *Nix*-833r/f PCR product sequences from individuals from four wild population samples (Guangzhou, Arco, Ban Rai, and Velika Gorica) and one sequence from the Chiang Mai pool. Dashes represent missing data and dots indicate identity to the reference sequence. (PDF 144 kb)
