# Supplementary material for: The Nix locus on the male-specific homologue of chromosome 1 in Aedes albopictus is a strong candidate for a male-determining factor
Source: Parasit Vectors. 2018 Dec 24;11(Suppl 2):647. doi: 10.1186/s13071-018-3215-8 (PMC6304787; doi:10.1186/s13071-018-3215-8)
Supplement: Supplementary file 1 — Table S1. Primer sequences. (DOCX 12 kb) [file 13071_2018_3215_MOESM1_ESM.docx]

Table S1. Primer sequences

| Primer | Sequence (5’ - 3’) | Melting temperature (°C) |
| --- | --- | --- |
|  |  |  |
| Nix-309f | TCGCTCAATTGGAATATTCGGA | 58 |
| Nix-309r | TGTATGATCACTGCGGTCCAT | 59 |
|  |  |  |
| Nix-833f | GGAAACATTCCCGCCGAAGT | 61 |
| Nix-833r | ACTGCGGTCCATTTCCTGTT | 60 |
|  |  |  |
| Nix-1121f | GCGTGTATGCTTTTTGGTTTGG | 60 |
| Nix-1121r | TCGAGAACCCTACGCCATTT | 59 |
|  |  |  |
| 18Sf | TGCCATGGATGCTTTCATTA | 60 |
| 18Sr | GTACAAAGGGCAGGGACGTA | 60 |
|  |  |  |
