# Supplementary material for: The Nix locus on the male-specific homologue of chromosome 1 in Aedes albopictus is a strong candidate for a male-determining factor
Source: Parasit Vectors. 2018 Dec 24;11(Suppl 2):647. doi: 10.1186/s13071-018-3215-8 (PMC6304787; doi:10.1186/s13071-018-3215-8)
Supplement: Supplementary file 3 — Figure S1. (PDF) Sequence of the Nix locus showing the positions of the exons (green box), intron and predicted amino acid sequence. The positions of the primers are shown. (PDF 76 kb) [file 13071_2018_3215_MOESM3_ESM.pdf]

```
                                      20                   40                   60                   80  NIX-1121f  100
NIX AAATATTAGTTTTATGACATACTTGTTTTCTGAGTGTAGCAAAAATATGAAAACACATTTTTGTACTTTGAATGTTAAGCGTGTATGCTTTTTGGTTTGG

                                     120                  140                  160                  180                  200
NIX TTATGTCAATATGTCAATTGTTAAACCCATGTTAATAGTTTTAATTTTTTTTAATCAAATTCTTTTTTTAAGTAATGTACAGTAAAAGTGAACTTAATCT
                                                                                     M  Y  S  K  S  E  L  N  L

                                     220                  240  NIX-833f            260                  280                  300
NIX CATTAACAATCAATTTGAATACATTAAAAAATATTGCATATACATTGGAAACATTCCCGCCGAAGTATCGAAAACAGATTTAATTGCAAAATTTTCCGTA
       I  N  N  Q  F  E  Y  I  K  K  Y  C  I  Y  I  G  N  I  P  A  E  V  S  K  T  D  L  I  A  K  F  S  V

                                     320                  340                  360                  380                  400
NIX TTTGGTGAAATATCTAACTTATACATGAAGTCATTCATTCAGTTTTGTGATGTGAAACCGGCAGTTGTTCGTTACAGACTGATGAAAAGTGTAAAGGAAT
      F  G  E  I  S  N  L  Y  M  K  S  F  I  Q  F  C  D  V  K  P  A  V  V  R  Y  R  L  M  K  S  V  K  E

                                     420                  440                  460                  480                  500
NIX CTTCAAGTTTACACAATAGTCGATATATTCAATCGGTTTTAATAGTTCTGCCACTAGATTCTTCCTACAATAATTACTTTCTTCCTTACAACACTTGTGT
    S  S  S  L  H  N  S  R  Y  I  Q  S  V  L  I  V  L  P  L  D  S  S  Y  N  N  Y  F  L  P  Y  N  T  C  V

                                     520                  540                  560                  580                  600
NIX TGTGGTATACACTTATAACAAATTTGGCATGGTAGATTTTTATCAAAAATTCAGTAAATTAGGAGATATACATGCGATGAAGAAAGCTACAAATGTCATG
       V  V  Y  T  Y  N  K  F  G  M  V  D  F  Y  Q  K  F  S  K  L  G  D  I  H  A  M  K  K  A  T  N  V  M

                                     620                  640                  660                  680                  700
NIX GTTTACATTAGCTTTGTATCAGAAAGAGCTGCAAGGACCATTCTGGATACTAAGCCTACAGATATACATATAAATGTACAAACAATTAATCATGTTACAC
      V  Y  I  S  F  V  S  E  R  A  A  R  T  I  L  D  T  K  P  T  D  I  H  I  N  V  Q  T  I  N  H  V  T

                                     720                  740                  760                  Nix-309f
NIX GAAATATTAACGTATGCTTAATAGATTTTGAAAAGGAATGTACATCAAATACGGCGATAAAATTAACACTTTTATATAATCGCTCAATTGGAATATTCGG
    R  N  I  N  V  C  L  I  D  F  E  K  E  C  T  S  N  T  A  I  K  L  T  L  L  Y  N  R  S  I  G  I  F  G

                                     820                  840                  860                  880                  900
NIX ACTACCATCTAATTTCACAGAAGCAAAACTGCACGATGAATTTTCAAGGTTTGTGTGGCAATGTGAATCCAAATGAGACAGATAAATATAAAAGCTACTC
       L  P  S  N  F  T  E  A  K  L  H  D  E  F  S  R

                                     920                  940                  960                  980                1,000
NIX AGATGAAAAGACCTTGAAGGTCGAAATTAGACATTATATTTTTATATGTTTTCAGGTATGGCAGAATTGAAAAAAATAGACTAGTGTACGACTCAACCGG
                                                                Y  G  R  I  E  K  N  R  L  V  Y  D  S  T  G

                                                                                   NIX-833r
                                                                                             Nix-309r
                                   1,020                1,040                1,060                1,080                1,100
NIX ACACTCTAAACAATACGGTTTTGTTTATTATGAAAAGCACTTGTCTGCTCAAGCGGCCAAACAGGAAATGGACCGCAGTGATCATACAGGACGTAAAATT
       H  S  K  Q  Y  G  F  V  Y  Y  E  K  H  L  S  A  Q  A  A  K  Q  E  M  D  R  S  D  H  T  G  R  K  I

                                   1,120                1,140                1,160                1,180      NIX-1121r 1,200
NIX GCAGTTCGGTTTGTTCCAGAAAAAGAGTAGGGCAGATATTAAATCTAGTGACAGAACAAAGCAACTAGCACCGTACTAAAAATGGCGTAGGGTTCTCGAA
      A  V  R  F  V  P  E  K  E  *

                                   1,220                1,240                1,260                1,280                1,300
NIX TATTTCGAGAGCTACTTACCATTTGTGTCATACACAATTAAAATGACTGACAAGTCTATCTACTTACTCGCAATCTATTTTATCGCAGGTTTATCAATAA

                                   1,320                1,340                1,360                1,380                1,400
NIX GAATTTTAGTGCCTGCACAACGTTGGGTTTGATCTGATGTAATAATCATAATCAAAATTTTCCAACCAAATTAAAGCTAGCTATATGTAAAGTAGCCTTG

                                   1,420                1,440                1,460                1,480                1,500
NIX CGTGAAACAAGTAACTTTTGCGTGATTTCATAAGCCCAAGGCTCAATTTAGCAATCAATGATTAGTACTACTATTAATATAACCATGTTAGGATCGGTTT

                                   1,520                1,540                1,560                1,580
NIX TAAATAACTAAATTTGTTGCTTCTGGTGAAAAACCATGTTTGAACTTTATGTTATATTTTGCTGTCGGGCTTAGTTACCG
```
