# Supplementary material for: The Nix locus on the male-specific homologue of chromosome 1 in Aedes albopictus is a strong candidate for a male-determining factor
Source: Parasit Vectors. 2018 Dec 24;11(Suppl 2):647. doi: 10.1186/s13071-018-3215-8 (PMC6304787; doi:10.1186/s13071-018-3215-8)
Supplement: Supplementary file 4 — Figure S2. (PDF) Alignment of the 12-24 h embryo-derived Nix transcript sequences showing a transcript with intron retention (unspliced) and a spliced transcript. The predicted translation products of the fragments are shown. (PDF 35 kb) [file 13071_2018_3215_MOESM4_ESM.pdf]

```
Spliced   CTACCATCTAATTTCACAGAAGCAAAACTGCACGATGAATTTTCAAG--------------------------------- 47
           L  P  S  N  F  T  E  A  K  L  H  D  E  F  S  R
Retained  CTACCATCTAATTTCACAGAAGCAAAACTGCACGATGAATTTTCAAGGTTTGTGTGGCAATGTGAATCCAAATGAGACAG 80
           L  P  S  N  F  T  E  A  K  L  H  D  E  F  S  R  F  V  W  Q  C  E  S  K  *

Spliced   --------------------------------------------------------------------------GTATGG 53
                                                                                     Y  G
Retained  ATAAATATAGAAGCTACTCAGATGAAAAGACCTTGAAGGTCGAAATTAGACATTATATTTTTATATGTTTTCAGGTATGG 160

Spliced   CAGAATTGAAAAAAATAGACTAGTGTACGACTCAACCGGACACTCTAAACAATACGGTTTTGTTTATTATGAAAGCACT 133
            R  I  E  K  N  R  L  V  Y  D  S  T  G  H  S  K  Q  Y  G  F  V  Y  Y  E  K  H
Retained  CAGAATTGAAAAAAATAGACTAGTGTACGACTCAACCGGACACTCTAAACAATACGGTTTTGTTTATTATGAAAGCACT 240

Spliced   TGTCTGCTCAAGCGGCCAAACAGGAA 159

Retained  TGTCTGCTCAAGCGGCCAAACAGGAA 266
           C  L  L  K  R  P  N  R
```
