# Supplementary material for: The Nix locus on the male-specific homologue of chromosome 1 in Aedes albopictus is a strong candidate for a male-determining factor
Source: Parasit Vectors. 2018 Dec 24;11(Suppl 2):647. doi: 10.1186/s13071-018-3215-8 (PMC6304787; doi:10.1186/s13071-018-3215-8)
Supplement: Supplementary file 5 — Figure S3. (PDF) Alignment of the 833 bp Nix fragment from the Rimini reference sequence with the 10 Nix-833r/f PCR product sequences from individuals from four wild population samples (Guangzhou, Arco, Ban Rai, and Velika Gorica) and one sequence from the Chiang Mai pool. Dashes represent missing data and dots indicate identity to the reference sequence. (PDF 144 kb) [file 13071_2018_3215_MOESM5_ESM.pdf]

```
Rimini reference ATCGAAAACAGATTTAATTGCAAAATTTTCCGTATTTGGTGAAATATCTAACTTATACATGAAGTCATTCATTCAGTTTTGTGATGTGAAACCGGCAGTT 100
Arco 1           -----------------------------....................................................................... 71
Arco 2           -----------------------------....................................................................... 71
Arco 3           -----------------------............................................................................. 77
Arco 5           --------------------------.......................................................................... 74
Guangzhou 2      -------------------------------..................................................................... 69
Guangzhou 4      ---------------------------------................................................................... 67
Guangzhou 5      .................................................................................................... 100
Ban Rai 4        -------------------------........................................................................... 75
Ban Rai 5        ------------------------------...................................................................... 70
Velika Gorica 1  -----------------------------....................................................................... 71
Chiang Mai       -----------------------............................................................................. 77

Rimini reference GTTCGTTACAGACTGATGAAAAGTGTAAAGGAATCTTCAAGTTTACACAATAGTCGATATATTCAATCGGTTTTAATAGTTCTGCCACTAGATTCTTCCT 200
Arco 1           .................................................................................................... 171
Arco 2           .................................................................................................... 171
Arco 3           .................................................................................................... 177
Arco 5           .................................................................................................... 174
Guangzhou 2      .................................................................................................... 169
Guangzhou 4      .................................................................................................... 167
Guangzhou 5      .................................................................................................... 200
Ban Rai 4        .................................................................................................... 175
Ban Rai 5        .................................................................................................... 170
Velika Gorica 1  .................................................................................................... 171
Chiang Mai       .................................................................................................... 177

Rimini reference ACAATAATTACTTTCTTCCTTACAACACTTGTGTTGTGGTATACACTTATAACAAATTTGGCATGGTAGATTTTTATCAAAAATTCAGTAAATTAGGAGA 300
Arco 1           .................................................................................................... 271
Arco 2           .................................................................................................... 271
Arco 3           .................................................................................................... 277
Arco 5           .................................................................................................... 274
Guangzhou 2      .................................................................................................... 269
Guangzhou 4      .................................................................................................... 267
Guangzhou 5      .................................................................................................... 300
Ban Rai 4        .................................................................................................... 275
Ban Rai 5        .................................................................................................... 270
Velika Gorica 1  .................................................................................................... 271
Chiang Mai       .................................................................................................... 277

Rimini reference TATACATGCGATGAAGAAAGCTACAAATGTCATGGTTTACATTAGCTTTGTATCAGAAAGAGCTGCAAGGACCATTCTGGATACTAAGCCTACAGATATA 400
Arco 1           .................................................................................................... 371
Arco 2           .................................................................................................... 371
Arco 3           .................................................................................................... 377
Arco 5           .................................................................................................... 374
Guangzhou 2      .................................................................................................... 369
Guangzhou 4      .................................................................................................... 367
Guangzhou 5      .................................................................................................... 400
Ban Rai 4        .................................................................................................... 375
Ban Rai 5        .................................................................................................... 370
Velika Gorica 1  .................................................................................................... 371
Chiang Mai       .................................................................................................... 377

Rimini reference CATATAAATGTACAAACAATTAATCATGTTACACGAAATATTAACGTATGCTTAATAGATTTTGAAAAGGAATGTACATCAAATACGGCGATAAAATTAA 500
Arco 1           .................................................................................................... 471
Arco 2           .................................................................................................... 471
Arco 3           .................................................................................................... 477
Arco 5           .................................................................................................... 474
Guangzhou 2      .................................................................................................... 469
Guangzhou 4      .................................................................................................... 467
Guangzhou 5      .................................................................................................... 500
Ban Rai 4        .................................................................................................... 475
Ban Rai 5        .................................................................................................... 470
Velika Gorica 1  .................................................................................................... 471
Chiang Mai       .................................................................................................... 477

Rimini reference CACTTTTATATAATCGCTCAATTGGAATATTCGGACTACCATCTAATTTCACAGAAGCAAAACTGCACGATGAATTTTCAAGGTTTGTGTGGCAATGTGA 600
Arco 1           .................................................................................................... 571
Arco 2           .................................................................................................... 571
Arco 3           .................................................................................................... 577
Arco 5           .................................................................................................... 574
Guangzhou 2      .................................................................................................... 569
Guangzhou 4      .................................................................................................... 567
Guangzhou 5      .................................................................................................... 600
Ban Rai 4        .................................................................................................... 575
Ban Rai 5        .................................................................................................... 570
Velika Gorica 1  .................................................................................................... 571
Chiang Mai       .................................................................................................... 577

Rimini reference ATCCAAATGAGACAGATAAATATAAAAGCTACTCAGATGAAAAGACCTTGAAGGTCGAAATTAGACATTATATTTTTATATGTTTTCAGGTATGGCAGAA 700
Arco 1           .................................................................................................... 671
Arco 2           .................................................................................................... 671
Arco 3           .................................................................................................... 677
Arco 5           .................................................................................................... 674
Guangzhou 2      .................................................................................................... 669
Guangzhou 4      .................................................................................................... 667
Guangzhou 5      .................................................................................................... 700
Ban Rai 4        .................................................................................................... 675
Ban Rai 5        .................................................................................................... 670
Velika Gorica 1  .................................................................................................... 671
Chiang Mai       .................................................................................................... 677

Rimini reference TTGAAAAAAATAGACTAGTGTACGACTCAACCGGACACTCTAAACAATACGGTTTTGTTTATTATGAAAAGCACTTGTCTGCTCAAGCGGCCA 793
Arco 1           ....................................................----------------------------------------- 723
Arco 2           ....................................................----------------------------------------- 723
Arco 3           ....................................................----------------------------------------- 729
Arco 5           ............................................................................................. 767
Guangzhou 2      .......................................................................---------------------- 740
Guangzhou 4      .....................................................---------------------------------------- 720
Guangzhou 5      ..................................................................--------------------------- 766
Ban Rai 4        .....................................................................------------------------ 744
Ban Rai 5        ...................................................------------------------------------------ 721
Velika Gorica 1  ....................................................----------------------------------------- 723
Chiang Mai       ....................................................----------------------------------------- 729
```
